# P3MC: A double blind parallel group randomised placebo controlled trial of Propranolol and Pizotifen in preventing migraine in children

**DOI:** 10.1186/1745-6215-11-71

**Published:** 2010-06-16

**Authors:** Paul Silcocks, Diane Whitham, William Patrick Whitehouse

**Affiliations:** 1 Nottingham Clinical Trials Unit, University of Nottingham, Queen's Medical Centre, Nottingham, NG7 2UH, UK; 2 University of Nottingham, E Floor East Block, Queen's Medical Centre, Nottingham, NG7 2UH, UK

## Abstract

**Background:**

A recent Cochrane Review demonstrated the remarkable lack of reliable clinical trials of migraine treatments for children, especially for the two most prescribed preventative treatments in the UK, *Propranolol *and *Pizotifen*.

Migraine trials in both children and adults have high placebo responder rates, e.g. of 23%, but for a trial's results to be generalisable "placebo responders" should not be excluded and for a drug to be worthwhile it should be clearly superior, both clinically and statistically, to placebo.

**Methods/Design:**

Two multicentre, two arm double blind parallel group randomised controlled trials, with allocation ratio of 2:1 for each comparison, Propranolol versus placebo and Pizotifen versus placebo. The trial is designed to test whether Propranolol is superior to placebo and whether Pizotifen is superior to placebo for the prevention of migraine attacks in children aged 5 - 16 years referred to secondary care out-patient settings with frequent migraine (2-6/4 weeks). The primary outcome measure is the number of migraine attacks during trial weeks 11 to 14.

**Discussion:**

A strength of this trial is the participation of clinically well defined migraine patients who will also be approached to help with future longer-term follow-up studies.

**Trial Registration:**

ISRCTN97360154

## Background

In the last 20 years the International Headache Society (IHS) has fostered the development of high quality research in headache including migraine. The 2004 (2^nd^) edition of the International Classification of Headache Disorders [1] provides a framework for headache research, including clinical trials. It is vital that trials use this classification which has become rather more child relevant in this last edition.

Inclusion of patients with Probable Migraine (PM), as defined in the classification, previously called "migraine-like headache" or "mixed headache" is vital for the trial to be of general use to most children presenting to paediatricians with severe headaches, because many general paediatricians will be unfamiliar with the precise operational criteria for Migraine subtypes.

A recent Cochrane Review [2] has demonstrated the remarkable lack of reliable clinical trials of migraine treatments for children, especially for the two most prescribed preventative treatments used in the UK, Propranolol and Pizotifen.

For a trial's results to be generalisable it should reflect the high placebo responder rates (around 23% [3]) typically found and not exclude "placebo responders". In addition for a drug to be worthwhile it should be clearly superior, both clinically and statistically, to placebo.

The clinical course of migraine is especially difficult to predict in children. Migraine will come for weeks, months or a few years then remit for months or years, sometimes returning unpredictably later on. Long term follow-up is difficult and studies have demonstrated this variability [4].

The aim of this trial is to confirm or refute superiority of Propranolol to placebo and of Pizotifen to placebo for the prevention of migraine attacks in children aged 5 - 16 years referred to secondary care out-patient settings with frequent migraine (2-6/4 weeks).

Both the active trial treatments or "Investigational Medicinal Products" (IMPs), Propranolol and Pizotifen have been in common clinical use for this indication in children for over 20 years. The trial does not therefore expose this group of patients to a new therapeutic risk, but will systematically evaluate efficacy and adverse events. The results will ascertain if one or both are superior to placebo in the prevention of migraine in children, and quantify other useful clinical outcomes such as quality of life, school attendance, any prolonged benefit after drug withdrawal, and adverse effects.

## Methods/Design

### Study design

Two simultaneous multicentre parallel group double-blind randomised placebo controlled trials of Propranolol and Pizotifen.

### Setting

Secondary care paediatric headache or neurology clinics.

### Participants

#### Inclusion criteria

1. age 5 years 0 months to 16 years 11 months

2. with Migraine with Out aura (MO), Migraine with Aura (MA), Probable Migraine (PM) as defined by IHS [1] (see Appendix E),

3. with 2 to 6 migraine or probable migraine attacks/4 weeks by history during the previous 3 months

4. and 2 to 6 migraine or probable migraine attacks/4 weeks during the 4 week run-in

5. and treating paediatrician and parent/guardian and child or young person believe the attacks are currently frequent and severe enough to merit a try of twice daily preventative medication

6. Satisfactory completion of headache diary during the run-in period at discretion of the investigator

#### Exclusion criteria

1. Asthma, bronchospasm or nocturnal or exercise induced cough or wheeze within the last 12 months or currently on daily asthma preventative treatment

2. children under paediatric cardiology review, at the discretion of their paediatric cardiologist, e.g. if Propranolol or Pizotifen were contraindicated

3. children with any of the following: uncontrolled heart disease, the presence of second or third degree heart block, in cardiogenic shock, bradycardia, severe peripheral arterial disease, metabolic acidosis, sick sinus syndrome, untreated phaeochromocytoma, prone to hypoglycaemia (e.g. after prolonged fasting) or Prinzmetal's angina.

4. previous severe adverse event probably related to Propranolol or Pizotifen

5. on Propranolol, another beta-blocker, Pizotifen or Cyproheptidine in the last 3 months

6. currently in or have been in another prospective drug trial in the last 3 months

7. fewer than 2 or more than 6 eligible attacks during the 4 week run-in, and stay excluded for 3 months at least

8. child or family unable to identify their migraine or probable migraine headaches confidently (as may happen with some patients with both mild headaches and migraine on different days, e.g. with chronic daily headache [15 or more headache days/month]).

9. females of child bearing potential who are not using a reliable contraceptive strategy such as abstinence, barrier methods, oral contraceptive pills and contraceptive injections. See Pregnancy section below.

10. Informed consent not given by parents/guardian, or assent/consent not given by patient

### Interventions

#### Propranolol

A non-selective beta-blocker which crosses the blood-brain barrier exerting central as well as peripheral effects and which has been used in migraine prevention since the 1960s [5]. It is generally well tolerated but in high dose can be associated with fatigue or sleep disturbance. It can also cause bronchospasm and exacerbate asthma.

#### Pizotifen

An antihistamine with histamine-1 antagonist and serotonin (5-HT2) antagonist properties that is structurally related to the tricyclic antidepressants. It has been used for over 20 years in the United Kingdom for migraine prophylaxis in children, young people and adults. It is generally well tolerated but can cause drowsiness, so it is commonly given as a once a day evening dose. Other adverse effects include increased appetite and weight gain.

#### Placebo

Both liquid and tablet formulations of the placebo will be manufactured using the same excipients used in the active formulation of the drugs minus the active ingredients. The placebo, Propranolol and Pizotifen tablets are matched in appearance; the liquid placebo matches the liquid Propranolol in appearance and taste but the liquid Pizotifen has a slightly different flavour.

All participants will be offered a choice of liquid or tablet preparations of the trial treatments. Because Pizotifen only has an evening dose, while Propranolol is given twice daily, to maintain blinding participants in the Propranolol and placebo arms will receive morning and evening doses from separate bottles; those in Pizotifen arm will take a morning placebo dose and an active evening dose (also from separate bottles).

For both active arms in this trial, starting, titration and age specific maintenance doses are consistent with the recommendations in "Medicines for Children" [6] and the new "British National Formulary (BNF) for children" [7] for 80% of participants by standard growth charts for the tablet preparations. The maximum dose of the liquid preparation is less to comply with World Health Organisation (WHO) recommendations on maximum intake of propylene glycol (used as a preservative for the liquid preparation of Propranolol).

### Concomitant therapy

#### Permitted medication

Any other regular medication (apart from Propranolol or other beta blocker, Pizotifen or Cyproheptidine in the 3 months before recruitment)

Other migraine preventative medication should normally be withdrawn first, but it may be continued as long as the dose does not change during the 12 week assessment.

#### Rescue medication and additional treatment(s)

All participants will be given an individual rescue treatment plan for migraine headaches, depending on their and their paediatrician's experience and preference. All rescue treatments used and their doses and effects will be recorded in the diary during the 4 weeks baseline assessment block 1 and the assessment blocks 2, 3 and 4 (weeks 11-14, 25-28, 37-40).

### Restrictions

Rizatriptan should be avoided by the trial participants while taking the trial treatments and for 5 days after stopping the trial treatments, because of a drug interaction with Propranolol.

Different non-steroidal anti-inflammatory drugs (NSAIDs) should not be used together. Aspirin should be avoided in children under 16 years. Use of any over-the-counter remedies will be checked by the research nurse.

#### Compliance

This will be assessed in 2 ways:

1) by verbally questioning the participant and parent/guardian at visits as to roughly how often a week a dose is missed. The % compliance is defined as 100 - % missed doses.

2) by examination of returned medication bottles, and measurement of the observed residual tablet numbers or residual liquid volumes. Missed doses by tablet number or volume will be expressed as:.

S = amount supplied,

R = amount returned

P = amount planned to be taken

The level of acceptable compliance with study medication will be set at >50% on both measures for the main outcome assessment period (weeks 11-14).

#### Criteria for terminating trial

The study may be stopped as a whole because of a regulatory authority decision, change in opinion of the REC or overwhelming evidence of efficacy/inefficacy, safety concerns or issues with trial conduct at the discretion of the Sponsor.

Recruitment at a centre may be stopped particularly for reasons of low recruitment, protocol violation or inadequate data recording.

### Hypotheses

#### Primary hypothesis

• To test whether Propranolol or Pizotifen are superior to placebo for the prevention of migraine attacks in children aged 5 - 16 years old with frequent migraine (2-6/4 weeks), who are referred to secondary care out-patient settings.

#### Secondary hypotheses

• To test whether any therapeutic effect out lasts the period of drug administration.

• To test whether a dose (in mg/kg/day) - response relationship exists at the doses used.

• To test whether active treatment improves participation by school attendance, and parent/guardian time off work, and health related and non-health related quality of life, and health status.

• To estimate cost-effectiveness if either active treatment proves superior to placebo.

### Sample size

The number of attacks per month is assumed to follow an over-dispersed Poisson distribution. A mean attack rate in the last month of treatment of 3 episodes with variance of 4 was assumed for the placebo arms based on the review of Victor & Ryan (2003) [2].

The sample size for the primary endpoint was estimated using formula 9.13 on page 176 of Machin et al [8] assuming a 33% reduction in the attack rate in the active arms. This formula gives the total number of attacks that must be observed based on a Poisson distribution, and this was divided by 2.5 (average number of attacks per person) to give the total number of participants in each study.

The over-dispersion in comparison with a Poisson distribution was allowed for by multiplying the required number of participants based on the standard formula by a factor of 1.33.

The sample size estimates for each trial assumed a power of 80% and 5% two-sided significance, with a 2:1 allocation of active: placebo treatment within each trial.

On these assumptions the required sample size is 226 evaluable participants for each trial, i.e. 452 in total, to detect a reduction in mean attack rate in both arms from 3 to 2 per month.

The target of 600 for recruitment also leaves a margin for drop out of up to 25% (=1-452/600) for the primary outcome but only 2% (=1-588/600) for the proportion of responders outcome.

### Randomisation and blinding

The randomisation will be based on a computer generated pseudo-random code using random permuted blocks of randomly varying size, created by the Nottingham Clinical Trials Unit (CTU) in accordance with their standard operating procedure (SOP) and held on a secure server. The randomisation proceeds in two stages: firstly a randomisation to one trial or the other; secondly a randomisation within each trial to active or placebo arm. The randomisation within each trial will be stratified by age (5-11 years *vs *12-16 years), type of migraine (two categories) and recruiting centre (10 centres).

Investigators will access the treatment allocation for each participant by means of a remote, internet-based randomisation system developed and maintained by the Nottingham CTU. The sequence of treatment allocations will be concealed until interventions have all been assigned and recruitment, data collection, and all other trial-related assessments are complete.

### Procedures and observations

#### Consent

The process for obtaining participant informed consent or assent and parent/guardian informed consent will be in accordance with the REC guidance, and Good Clinical Practice (GCP) and any other regulatory requirements that might be introduced.

Potential participants will be identified in clinic based on their clinical diagnosis and history of migraine or probable migraine attack frequency for the previous 3 months. If the potential participant and their parent/guardian are willing to participate but cannot estimate the migraine attack frequency in the previous 3 months they will be given a standard headache diary to complete over the next 3 months, and appropriate advice and treatment will be given as is normal practice.

In the event of their withdrawal data collected so far will not be erased and will be used in the final analyses where appropriate (this will be explained in the Participant and Parent/Guardian Information Sheets).

### Baseline measurements

1. A standard headache diary will be completed by the participant and their parent/guardian. This is adapted for the trial from a migraine clinic diary developed by the British Paediatric Neurology Association's (BPNA's) Governance & Audit group [9].

2. Headache intensity scale [10] - a four point self-rated scale (assisted if needs be by the parent/guardian). This is the functionally based scale recommended by the IHS to assist in diagnosis, monitoring treatment clinically and in trials.

3. Pediatric Migraine Disability Assessment Scale (PedMIDAS), a standardised validated health-related quality of life scale for children with migraine [11].

4. Generic Child Quality of Life Measure (GCQ10), a standard validated non-health related quality of life scale for children [12].

5. EQ-5D [13], a standardised validated health outcome measure providing a simple descriptive profile and a single index value for health status. For parents/guardians and participants aged 12-16 years old.

6. Child-friendly EQ-5D [14] For younger participants; a child friendly version of EQ-5D has been developed and will be used during the trial for participants aged 7-11 years.

7. UK Proxy EQ-5D [15]

8. Age, sex, height, weight, blood pressure and heart rate at baseline and at all clinical visits

9. Parent's/guardian's stage at leaving full-time education

10. Full post code (to derive deprivation score for area of residence).

11. Office of National Statistics (ONS) self-coded NS-SEC of one parent/guardian [16]

### Outcome measurements

#### Primary outcome

The number of migraine attacks during weeks 11 to 14 from randomisation, as recorded in the participant diary, with an attack being defined as in the IHS International Classification of Headache Disorders [10].

#### Secondary outcomes

##### Efficacy

1. **Response**, defined as a 50% or greater reduction in number of attacks during weeks 11 to 14; 25 to 28; 37 to 40, relative to baseline, as recorded in the participant diary.

2. **Headache intensity; **headache intensity will be measured on the standard 4 point scale [10]. As migraine headache is usually moderate or severe (with additional features), mild headaches will not be recorded, unless they are associated with sufficient additional features to meet the diagnostic criteria for migraine attack. Headache intensity as experienced during the worst part of the migraine attack will be averaged during weeks 11 to 14 for each participant.

3. **Use of rescue medication**, defined as the number of rescue doses taken during weeks 11 to 14, as recorded in the participant diary.

4. **School attendance**, defined as % of school half days attended during weeks 11 to 14, as recorded by diary.

5. **Recalled Attack Frequency**: participants reply at visit 6 to "How many migraine attacks did you have in the 4 week assessment block (weeks 11 to 14)?" They may discuss with their parents/guardian and look at their diary if they wish. This will help in imputing missing values for those who can provide no diary information.

6. Quality of life & functional outcomes using:

6.1 the health-related quality of life tool PedMIDAS;

6.2 the non-health-related Generic Child Quality of Life measure GCQ.

##### Health Economics

Sufficient data to allow cost effectiveness comparisons from NHS and family perspectives will be collected but not analysed at this stage. If one or other active treatments proves effective in comparison to placebo, then separate funding for a formal cost comparison and cost effectiveness study will be sought.

1. Parent's/guardian's time off work mainly related to child's migraine, for those in full time paid employment, or pro-rata for those in part-time paid employment, during weeks 11 to 14, as recorded in the participant diary.

2. Costs of Propranolol, Pizotifen, and placebo (study medications)

3. Cost of rescue medications

4. Number and length of emergency hospital admissions and Emergency Department attendances, and non-trial hospital and GP surgery appointments, related to migraine, with dates and place will be recorded in the participant diaries so the cost of investigations can be determined later

5. Cost of "child half days off school" (4 above)

6. EQ-5D for parents/guardian and participants aged 12-16 years; UK Proxy EQ-5D for younger participants.

#### Adverse events

Safety and tolerability variables

These include:

1. Adverse Event (AE) reports:

a) spontaneous,

b) elicited by routine enquiry using the AE check list,

c) findings from physical and neurological examinations

2. Vital signs: Blood Pressure, Heart Rate, Weight and Height.

3. Participant's wish to continue (or parent/guardian's where child unsure) with allocated trial medication after week 14.

4. Time until withdrawal from allocated trial medication, from randomisation to end of week 14. Reasons for withdrawal will be recorded in CRF.

#### Follow up procedures

**Figure 1:**
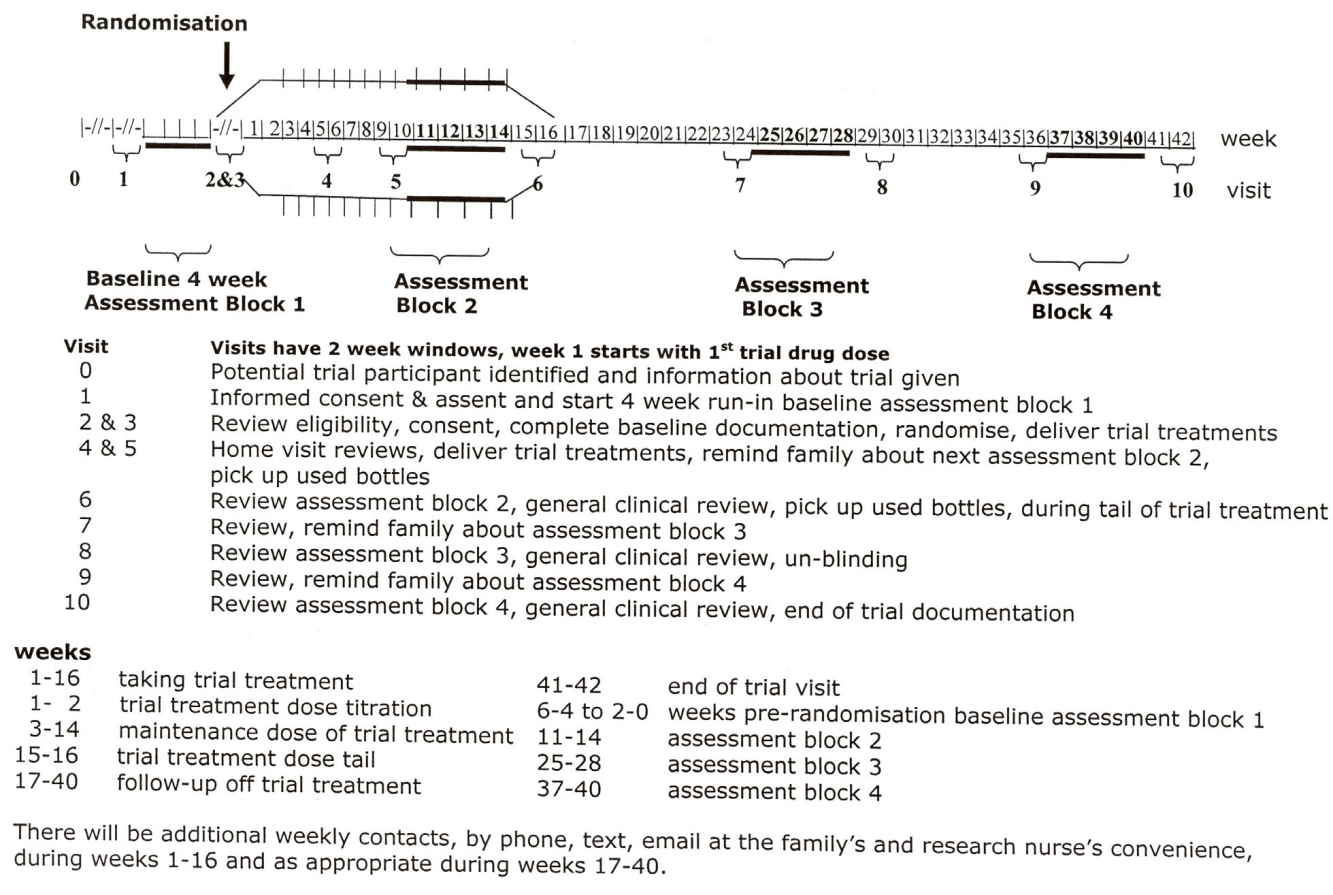
**Patient and family involvement over time**.

Participants in all three groups will follow the same schedule of study visits up to the end of the trial, regardless of their compliance with the trial medication. Screening & baseline assessments will be performed during visits 1-3, with a 4 week "run-in" ending in randomisation at visit 2 and start of trial treatment at visit 3. Visits 4-6 will take place during the 2 week dose escalation period, 12 week maintenance and 2 week down-titration phases. Visits 7 & 8 will take place during a 3 month blinded off-treatment phase, visit 9 during a 3 month unblinded follow-up with visit 10 marking the end of the Trial, at which point there will be an option to consent for a possible future follow-up study, (Figure 1).

#### Data Monitoring Committee (DMC)

An independent Data Monitoring Committee (DMC) will evaluate the outcome and safety data in the context of the overall trial and the currently existing information about the study drugs. No formal interim analyses for efficacy are planned. For these "administrative" analyses, informal Haybittle-Peto type boundaries [17,18] will be adopted for efficacy to permit the DMC to break the blind if it wishes, with negligible effect on the properties of the final analysis

#### Types of Analyses

The primary efficacy parameter will be the relative attack rate between the two treatment arms and their placebo arms, estimated by a Poisson regression model. The model will include terms to account for treatment arm, stratification variables and other covariates (baseline frequency of attacks, prior Triptan use and whether treatment naïve). The anticipated over- dispersion will be accounted for by estimation of robust standard errors.

The analysis will be performed on the full analysis set (following the intention to treat principle). For the primary efficacy analysis the statistical test will be two-sided at a nominal 5% two-sided significance level (see sample size justification).

##### Secondary outcomes

All secondary endpoints will be analysed using analysis of covariance, logistic regression or Poisson regression as appropriate. Secondary analyses will include a repeated measures analysis of the attack frequency at different time points (11-14 weeks; 25-28 weeks; 37-40 weeks from randomisation). Headache frequency will also be analysed with respect to the mean mg/kg/day dose for the active treatments for each participant during this period.

Again, terms to account for treatment arm, stratification variables and covariates will be included in the model with allowance for over-dispersion of binary/count data. Time to event data will be handled by survival regression. All secondary endpoints will be analysed only in the full analysis set.

For the secondary analyses (excluding cost analyses), tests and confidence intervals will also be two-sided and performed at the 5% significance level. No adjustment for multiple testing will be performed.

#### Adverse events

All adverse events will be listed. Treatment-emergent AEs (defined as AEs which first develop or which worsen after the start of trial treatment) will be summarised by treatment, severity and relationship to treatment.

In addition the frequency (number of AEs and number of patients experiencing an AE) of treatment-emergent AEs will be summarised using the Medical Dictionary for Regulatory Activities [19] (MedDRA v 9.1 or later) by primary body system and preferred term.

Physical and neurological findings at baseline and any changes occurring during treatment will be listed.

Vital signs will be listed and summarised, together with changes from baseline.

#### Treatment compliance

Treatment compliance will be summarised by treatment group and time interval since randomisation.

#### Success of blinding

This will be assessed in participants, parents/guardian and investigators by a two-part question at the week 29-30 visit, just prior to un-blinding. The first part will ask whether the participant was believed to have received an active treatment or placebo. The second question will be asked only if the answer to the first question was "active", and will ask which active treatment was thought to have been given. Responses to both questions will include a "don't know" category, and the analysis will correct for guessing.

#### Procedures for missing data

A major goal of this study is to obtain virtually complete follow-up. The research nurses will ensure this as far as possible by home visits and close telephone/texting/email contact. No missing values are expected for the key baseline covariates because these data must be submitted prior to randomisation.

Missing covariate and response values will be handled by multiple imputation using chained equations, by means of the Stata add-in module *ice *[20]. In particular the imputation for missing response data during the final 4 weeks will incorporate information on earlier baseline response data and other variables thought likely to account for the missing data. A sensitivity analysis in which missing outcome data are assumed to be missing not at random will also be performed for the primary outcome and for response.

### Ehtical approval

The protocol has been given full ethical approval by the Trent Research Ethics Committee (Reference: 09/H0405/19). It is fully compliant with the Helsinki Declaration.

## Discussion

The protocol design posed some particular challenges. The funding brief was for a 3 arm trial of Propranolol, Pizotifen, and Placebo. For statistical and operational reasons we are technically undertaking 2 parallel 2-arm trials, but blinding will make it indistinguishable to participants and local investigators from a 3 arm trial. This way if there is a problem with one trial the other can continue un-hindered.

We undertook several consumer involvement exercises, with help from the Medicines for Children Research Network (MCRN), and it was clear that keeping participants and their parents/guardians blinded for up to 2 years after their participation was not acceptable and could present a significant barrier to recruitment and retention. Participants and their families were content to be blinded during the trial treatment phase, but wanted to know what the treatment had been as soon as possible afterwards, to inform future treatment decisions. We were keen to maintain the blinding for as long as possible to avoid investigators being biased in their approach to potential participants, e.g. by being biased by preliminary results against one of the active treatments. Also we felt that the long-term assessments would be compromised if they were undertaken unblinded. As a compromise, and taking note of consumers concerns, we decided to unblind at visit 8 (week 29/30) see Figure 1. Consumers at the focus group were supportive of this delay in unblinding.

We were keen to develop a protocol particularly suited to children and young people. In contrast to adult participants, they often express fear and avoidance of medical settings and procedures, although they are usually curious and supportive of clinical research in general and are often remarkably altruistic. With this in mind we minimised the invasive procedures commonly undertaken in trials: there are no blood tests and only routine out-patient procedures. Most contacts with the participants will be by home visits by research nurses, or by phone call, with hospital visits approximately every 3 months, so that approximately 1 additional visit over the year is anticipated. We needed a liquid formulation, as younger children would be unable to swallow tablets. However, we decided to offer participants the choice of liquid *or *tablets as our consumer involvement work suggested that many young people of secondary school age (teenagers) did not want to take liquid preparations, and that would prove a barrier to recruitment. Tablet and liquid formulation concentrations were such that the numbers of tablets taken were the same for Propranolol, Pizotifen, placebo, during titration, maintenance and withdrawal, phases as were the volumes of liquid formulations.

Being able to offer participants tablets also mitigates the problem posed by the maximum ceiling dose caused by the propylene glycol (see "interventions section above) in the Propranolol liquid. We anticipate that most participants will take tablets because migraine is more common in secondary school aged young people than younger children.

We considered blinding essential in this definitive study, and ideally wanted full blinding of participants, their parents/guardian, their paediatrician/local investigator and research nurse, and trial statistician, for which trial the participants were in (Propranolol, or Pizotifen), as well as within each trial (active *vs *placebo). To avoid sleepiness commonly seen with Pizotifen we decided that Pizotifen would only be given at bedtime (which is frequent routine clinical practice), and to keep the blind a placebo dose is given in the mornings. Families therefore are given a supply of morning doses as well as a supply of evening doses, and the importance of using the correct supply at the correct time will be emphasised by the research nurses.

Also rather than over encapsulation we opted to manufacture identically looking tablets of Propranolol, Pizotifen and placebo, as we feared children or their parents would deliberately unblind themselves out of curiosity, or by accident. If this were to happen in a significant proportion of participants the trial could be undermined. However, manufacturing tablets created other problems and increased costs and delays.

Propranolol and Pizotifen have been widely used for migraine in children for many years and so the exposure in this trial is relatively low risk.

We are also making a special effort to allow for effects of missing values of the primary response variable and covariates through use of multiple imputation.

## List of abbreviations

AE: Adverse Event; BASH: British Association for the Study of Headache; BNF: British National Formulary; BPNA: British Paediatric Neurology Association; CRF: Case Report Form; CTU: Nottingham Clinical Trials Unit; DMC: Data Monitoring Committee; GCQ: Generic Children's Quality of Life Scale; HTA: Health Technology Assessment programme; IHS: International Headache Society; ISRCTN: International Standard Randomized Controlled Trial Number Register; MA: Migraine with Aura; MedRA: Medical Dictionary for Regulatory Activities; MHRA: Medicines and Healthcare products Regulatory Agency; MO: Migraine without aura; NSAIDs: Non-steroidal anti-inflammatory drugs; ONS: Office of National Statistics; PedMIDAS: Pediatric Migraine Disability Assessment Questionnaire; P/GIS: Parent/Guardian Information Sheet; PIS: Participant Information Sheet; PM: Probable Migraine; REC: Research Ethic Committee; SOP: Standard Operating Procedure; WHO: World Health Organisation

## Competing interests

The authors declare that they have no competing interests.

## Authors' contributions

WW conceived the study, is Chief Investigator, participated in the design and helped draft the manuscript. PS participated in the design of the study, drafted the manuscript and is the trial statistician. DW participated in the study design, reviewed the protocol, helped draft the manuscript and is responsible for trial set-up and coordination. All authors have read and approved the final manuscript.
